# A novel germline mutation of PTEN associated with brain tumours of multiple lineages

**DOI:** 10.1038/sj.bjc.6600206

**Published:** 2002-05-03

**Authors:** F J T Staal, R B van der Luijt, M R M Baert, J van Drunen, H van Bakel, E Peters, I de Valk, H K P van Amstel, M J B Taphoorn, G H Jansen, C W M van Veelen, B Burgering, G E J Staal

**Affiliations:** Department of Immunology, Erasmus University Rotterdam, Rotterdam, the Netherlands; Department of Medical Genetics, University Medical Center, Utrecht, the Netherlands; Department of Neuro-oncology and Neurosurgery, University Medical Center, Utrecht, the Netherlands; Department of Pathology, University Medical Center, Utrecht, the Netherlands; Department of Physiological Chemistry, University Medical Center, Utrecht, the Netherlands

**Keywords:** PTEN, tumour suppressor gene, glioma, transfection, PKB, Akt

## Abstract

We have identified a novel germline mutation in the PTEN tumour suppressor gene. The mutation was identified in a patient with a glioma, and turned out to be a heterozygous germline mutation of PTEN (Arg234Gln), without loss of heterozygosity in tumour DNA. The biological consequences of this germline mutation were investigated by means of transfection studies of the mutant PTEN molecule compared to wild-type PTEN. In contrast to the wild-type molecule, the mutant PTEN protein is not capable of inducing apoptosis, induces increased cell proliferation and leads to high constitutive PKB/Akt activation, which cannot be increased anymore by stimulation with insulin. The reported patient, in addition to glioma, had suffered from benign meningioma in the past but did not show any clinical signs of Cowden disease or other hereditary diseases typically associated with PTEN germline mutations. The functional consequences of the mutation in transfection studies are consistent with high proliferative activity. Together, these findings suggest that the Arg234Gln missense mutation in PTEN has oncogenic properties and predisposes to brain tumours of multiple lineages.

*British Journal of Cancer* (2002) **86**, 1586–1591. DOI: 10.1038/sj/bjc/6600206 www.bjcancer.com

© 2002 Cancer Research UK

## 

PTEN, also designated as MMAC1 or TEP1, is a tumour suppressor gene located on chromosome 10q23 (Li *et al*, 1997; Bonneau and Longy, 2000). Somatic PTEN mutations have been identified in a variety of human cancers including breast, prostate, thyroid and endometrial carcinomas, malignant melanomas and gliomas (Bonneau and Longy, 2000). Mutation of PTEN appears to occur early in tumour development in endometrial cancer but late in the development of gliomas. Germline mutations in PTEN have also been found in the dominantly inherited Cowden, Lhermitte-Duclos, and Bannayan-Zonana syndromes, which are characterised by the formation of multiple benign tumours (Nelen *et al*, 1997).

Disruption of PTEN in the mouse results in early embryonic lethality. PTEN heterozygous mice display hyperplastic features as well as high tumour incidence suggesting that inactivation of PTEN plays important roles in tumorigenicity (Di Cristofano *et al*, 1998; Suzuki *et al*, 1998; Podsypanina *et al*, 1999). The PTEN gene contains nine exons and encodes a 403-aa protein, which possesses phosphatase activity on phosphotyrosyl and phosphoseryl/threonyl residues (Li and Sun, 1997; Bonneau and Longy, 2000). PTEN is an inefficient protein phosphatase *in vitro* but is very active on highly acidic substrates. The main PTEN substrate has proven to be phosphatidyl inositol (3,4,5)-triphosphate (PIP-3), which is the direct product of PI-3-kinase (Maehama and Dixon, 1998).

It has been proven that cells lacking wild-type PTEN from gliomas are characterised by elevated levels of PIP-3 (Myers and Tonks, 1997). As a consequence, the activity of protein kinase B (PKB/Akt) was found to be elevated, resulting in prevention of apoptosis (Stambolic *et al*, 1998). Introduction of wild-type PTEN reduced the levels of PIP-3 and protein kinase B (Li and Sun, 1998). In addition, wild-type PTEN was found to suppress the proliferation and the tumour growth of PTEN deficient glioblastoma cells, whereas mutant phosphatase inactive PTEN failed to suppress cell growth (Cheney *et al*, 1998; Furnari *et al*, 1998). Thus, two biological processes, namely cell proliferation and apoptosis are partly regulated by PTEN activity.

In searching for PTEN mutations in human gliomas we detected a heterozygous mutation (Arg234Gln) of PTEN in a patient with a glioma. The mutation was present in tumour DNA, as well as in DNA isolated from peripheral blood lymphocytes. No signs of Cowden disease were found in the patient, nor did we find any indications for loss of heterozygosity (LOH) of PTEN in tumour material. The biological consequences of this germline mutation have been evaluated by means of transfection studies of the mutant PTEN molecule compared to wild-type PTEN. Our results suggest that this mutation plays a role in regulating apoptosis and cell proliferation.

## PATIENT AND METHODS

### Patient and histology

In 1981 a 38-year old male, with an unremarkable medical history and with no family history of brain tumours, presented with focal seizures of the right arm and dysphasia. No neurological abnormalities were found on examination. Anti-epileptic drug medication resulted in a marked reduction of the seizures. In 1985 a CT brain scan was made because of progressive headaches and memory difficulties. A large right frontal lesion was found suggestive of a meningioma; this was removed completely. The diagnosis of meningioma was confirmed by the pathologist, but there were no signs of malignancy. In 1990 the seizures spread to the patient's right leg.

A CT scan revealed a non-enhancing lesion with slight mass effect in the left frontal lobe; a low grade glioma was suspected. The patient's symptoms progressed and he underwent surgery for this lesion in 1993. Histologic examination revealed tumour cells with peri-nuclear unstained cytoplasm, with irregular round to oval nuclei, with some mitotic activity (three per square mm). The capillary network in the tumour was unremarkable and there was no necrosis. This tumour was classified as an anaplastic oligodendroglioma. The patient underwent radiotherapy.

In 1998 the patient suffered a decline of cognitive functioning. A new CT brain scan showed regrowth of the left frontal tumour, which was again treated surgically. Histologic examination revealed a similar profile to that in 1993, but with more pleiomorphism of the nuclei of the tumour cells, more mitotic activity (eight per square mm), microvascular proliferation, and small areas of necrosis. The diagnosis was an anaplastic oligodendroglioma with signs of ongoing de-differentiation.

He was treated with chemotherapy for residual tumour, but after nearly a year there was further tumour growth. His clinical condition is steadily deteriorating.

### Tumour sample

Tumour samples were collected in the Department of Neurosurgery, University Medical Center, Utrecht, the Netherlands. Immediately after removal, parts of the tumour were snap-frozen in liquid nitrogen and stored at −80°C until required.

### DNA extraction

Tumour tissue was cut into 10 μm thick sections and mounted onto glass slides. Reference slides were stained with haematoxylin and eosin and examined by the pathologist in order to grade the tumour according to WHO criteria and for selecting tumour regions for analysis. The marked slide was used to guide microdissections. DNA extraction was carried out with the use of proteinase K and phenol/chloroform. High molecular weight DNA of the patient was also isolated from peripheral blood according to established procedures.

### LOH analysis

For LOH analysis, a total of six highly polymorphic markers from the PTEN region were used: five microsatellite markers flanking PTEN (D10S1645, D10S579, D10S215, D10S603 and D10S541) and the intragenic marker D10S2491. PCR reactions were performed in a GeneAmp 9600 PCR System (PE Biosystems) in a 10 μl volume containing 30 ng genomic DNA as template, 25 ng of each oligonucleotide primer, 200 μM of each dNTP and 0.4 unit AmpliTaq Gold DNA polymerase (Perkin Elmer), in 1× AmpliTaq Gold buffer with 2.5 mM MgCl_2_. An initial denaturation of 5 min at 95°C was followed by 36 cycles of 30 s at 95°C, 30 s at 55°C, 30 s at 72°C, and a final extension step of 10 min at 72°C. Markers were analysed on an ABI Prism™ 377 DNA Sequencer (PE Biosystems) using GeneScan and Genotyper software.

### Sequence analysis

Direct sequencing of the nine PTEN exons and their immediately flanking regions was performed using the oligonucleotide primers published by Steck *et al* (1997). PCR reactions were performed in a GeneAmp 9600 PCR System (PE Biosystems) in a 50 μl volume containing 100 ng genomic DNA as template, 50 ng of each oligonucleotide primer, 200 μM of each dNTP and 1.25 unit AmpliTaq Gold DNA polymerase (Perkin Elmer), in 1×AmpliTaq Gold buffer with 2.5 mM MgCl_2_. An initial denaturation of 10 min at 95°C was followed by 35 cycles of 30 s at 95°C, 30 s at 55°C, 1 min at 72°C, and a final extension step of 10 min at 72°C. For the amplification of exons 1 and 2, the annealing temperature was 57 and 50°C, respectively. Sequencing was performed on an ABI Prism™ 377 DNA Sequencer (PE Biosystems) using the Rhodamine Terminator Cycle Sequencing Ready Reaction Kit, according to the manufacturer's instructions.

### Constructs, transfections and proliferation assays

Wild-type and Arg234Gln mutant PTEN were cloned into the pCDNA3 expression vector by using PCR primers containing restriction sites. For wild-type PTEN the forward primer contained a *Bam*HI site, the reverse primer a *Xho*I site. The mutant site was introduced by means of two partially overlapping primers containing the mutation and introducing an *Eco*RI site. The *Eco*RI site was used to confirm the presence of the mutation in the final construct.

Sequences: PTEN FW: 5′ CAAGGATCCACCATGACAGCCATCATCAAAGAG 3′

PTEN RV: 5′ CGTTGCCTCGAGTCAGACTTTTGTAATTTGTGTATG 3′

MUT PTEN FW: 5′ GCCTGAATTCGAGGAATATATCTTCACCTTTAG 3′

MUT PTEN RV: 5′ TTCCGAATTCAGGACCCACACGACAGGAAGACAAGTTCATGTACTTC 3′.

PCR was performed in a 50 μl volume containing 100 ng pSG5-PTENwt as template, 50 ng of each oligonucleotide primer, 200 μM of each dNTP and 1.25 unit AmpliTaq Gold DNA polymerase (Perkin Elmer), in 1×AmpliTaq Gold buffer with 2.5 mM MgCl_2_. An initial denaturation of 10 min at 95°C was followed by 25 cycles of 30 s at 95°C, 30 s at 57°C, 1.25 min at 72°C, and a final extension step of 7 min at 72°C. PCR products were separated on a 1% agarose gel, purified, and digested with *Xho*I and *Bam*HI (wt), or the two PCR products for mutant PTEN with *Bam*HI and *Eco*RI and with *Eco*RI and *Xho*I. Wt PTEN was cloned into *Bam*HI/*Xho*I digested pCDNA3 by direct ligation, and the mutant PTEN by three-point ligation. Integrity of the wild-type and mutant forms was confirmed by sequencing the full inserts, as above. For transfections, the plasmids were digested with *Xba*I, in order to linearise them outside the neomycin resistance cassette and outside the PTEN coding sequence.

U87 glioma cells, which lack functional PTEN were transfected with the pcDNA expression constructs (which contains a neomycin resistance gene) using Fugene-6 (Boehringer Mannheim), according to the manufacturer's instructions. Two days after transfection, cells were put under selection using G418 (1.0 mg/ml). After approximately 3 weeks of selection, stable selected cells were checked for cell proliferation by seeding 5000 cells from the stable transfectants and manual counting at days 4, 6, 8 and 11.

### Western blotting

Equal numbers of U87 cells were lysed in boiling SDS sample buffer, loaded on a 10% polyacrylamide gel, separated by electrophoresis and blotted onto nitrocellulose by standard methods. PTEN protein was revealed using the anti-PTEN Antibody 9522 (Cell signalling Technology) and a Goat anti-rabbit-HPRO second step antibody, followed by visualisation by enhanced chemiluminescence (ECL, Amersham). For PKB assays, U87MG cells were seeded and grown to subconfluency, serum-starved for 18 h, and either left untreated or treated with insulin for 10 min then washed with PBS twice and directly harvested in Leamlli sample buffer. Fifty μg of protein was analysed by SDS–PAGE for phosphorylation of PKB by immunoblotting using an antibody against phosphorylated serine 473. The phosphorylation of this residue activates PKB/Akt.

### Apoptosis assay

Expression plasmids that target PTEN to the cell membrane using the CAAX motif were constructed in pcDNA by standard methods. Apoptosis assays were done as described (Kalejta *et al*, 1997). The CAAX constructs or empty vector (18 μg) were transfected by electroporation (270 V; 960 uF) into BaF3 promyelocytic cells, together with a Spectrin-GFP plasmid (2 μg). Two hours after transfection viable cells were collected by Ficoll density centrifugation, and cultured for another 24 h. The next day cells were harvested and resuspended in 300 μl PBS to which 700 μl ice cold 100% ethanol was added. Cells were incubated at −20°C for 2 h, washed and incubated with a propidium iodide/RNAse solution for 30 min at 37°C and put on ice in the dark till analysis on a FACScan flow cytometer. The number of transfected apoptotic cells was enumerated by electronic gating on GFP+ cells, and generating a cell cycle profile for these cells. The percentage of sub 2n cells was taken as a measure for the percentage of apoptosis.

## RESULTS

### Mutation analysis and LOH

**Figure 1:**
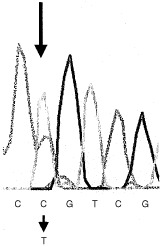
Identification of an Arg234Gln germline PTEN mutation in a patient with multiple brain tumours. A partial sequence of exon 7, obtained from tumour (glioma) DNA is shown. In the sequence (which is in reverse orientation), the single base change (C to T) is indicated by an arrow. The heterozygous peak corresponds to a G to A substitution at position 701 in the PTEN coding sequence, and results in an arginine to glutamine amino acid change at codon 234. Note the heterozygous state of the missense mutation, suggesting that there is no LOH of PTEN in the tumour.

The PTEN gene from a patient with glioma was analysed. PCR of all nine exons of the PTEN gene followed by sequencing revealed a G to A (CGACGG→CGACAG) substitution at position 701 (PTEN cDNA sequence U93051, GenBank) in exon 7, changing an arginine (Arg) residue to a glutamine (Gln) at codon 234. Figure 1

depicts the sequence analysis showing the missense mutation detected. The Arg234Gln (R234Q) mutation is located at the surface of the protein of the so-called C-2 domain (Lee, 1999). The mutation was found in DNA obtained from peripheral blood, the meningioma and the two oligodendrogliomas of the patient. The mutation has not been observed in 80 normal control chromosomes (data not shown), therefore it is unlikely to represent a common polymorphism.

Taken together, this therefore indicates that the R234Q mutation is a pathogenic germline mutation. No family studies could be carried out. To determine whether the PTEN mutation detected was associated with loss of heterozygosity in chromosome 10q, microsatellite analysis was carried out using markers mentioned in Materials and Methods. No LOH could be detected, which is in agreement with the heterozygous nature of the mutation in tumour material observed by DNA sequencing.

### Cell proliferation analysis

**Figure 2:**
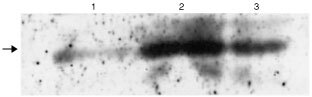
Western blot of stably transfected U87-MG glioma cells. The U87 glioma cell has a dysfunctional PTEN molecule and was transfected with R234Q mutant (lane 2) or wild-type (lane 1). Stable transfectants express higher levels of PTEN than the parental line (lane 3). The 54 kD PTEN band is indicated by an arrow.

In order to functionally characterise the consequence of the R234Q mutation identified, we performed two different assays concerning two processes, in which PTEN has been shown to play a key role, namely cell proliferation and apoptosis. We cloned wild-type and mutant PTEN into an expression plasmid and stably transfected U87MG glioma cells that lack functional PTEN. In this cell line, the PTEN gene carries a mutation in the splice donor site of intron 3, resulting in deletion of exon 3 in the mature transcript, leading to an unstable, nonfunctional protein that can only be detected at low levels. Western blot analysis revealed that PTEN levels in the transfectants were roughly equal to each other (Figure 2

) and similar to cell lines that contain functional PTEN (not shown). In contrast, the U87 mother cell line contains lower levels of (non-functional) PTEN compared to the transfectants (Figure 2).

**Figure 3:**
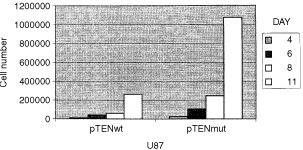
R234Q Mutant PTEN allows higher levels of proliferation than wild-type PTEN. The stable U87 transfectants were grown under neo-selection and 5000 cells were seeded on day 0. Pints cells were harvested and counted at the indicated time.

The wild-type and mutant PTEN transfectants were compared for their growth properties. By counting the number of cells over a 2-week period, it became apparent that the mutant cells proliferated at a roughly five-fold higher rate than cells transfected with wild-type PTEN (Figure 3

). Thus, the R234Q mutation in PTEN that we found permits cells to proliferate at a much higher rate.

### Apoptosis

**Figure 4:**
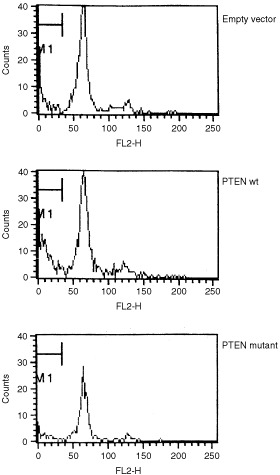
Mutant PTEN does not induce apoptosis, whereas wild-type does. BAF/3 cells were transfected with Spectrin-GFP, together with empty vector control (A) CAAX-PTEN wild-type (B), CAAX-PTEN mutant (C). Cell cycle profiles for propidium iodide stained cells, electronically gated on GFP+ cells are shown.

The wild-type and mutant PTEN were cloned into a so-called CAAX vector to target them to the cell membrane. Using BaF3 promyelocytic cells, which are IL-3 dependent and sensitive to apoptosis, we investigated the consequence of these forms of PTEN for apoptosis. In order to identify transfected cells, the PTEN molecules were co-transfected with a spectrin-GFP construct. Gating on GFP positive cells thus permitted investigating only transfected cells. As shown in Figure 4

, cells transfected with an empty vector (together with Spectrin-GFP), have approximately 18% apoptotic cells. Transfection of wild-type PTEN almost doubles the percentage of apoptotic cells (32%), in contrast mutant PTEN does not induce any apoptosis (16% of sub 2n cells) and in some experiments the percentage of apoptotis after transfection of the mutant was even lower than in cells transfected with Spectrin-GFP alone.

### PKB/Akt activity

**Figure 5:**
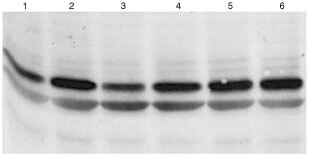
Increased constitutive PKB activity, but lack of insulin-induced PKB activity in R234Q PTEN transfectants. U87MG transfected wild-type cells (lanes 1 and 2), U87MG cells transfected with normal PTEN (lanes 3 and 4) and mutant PTEN (lanes 5 and 6) were seeded and grown to subconfluency, serum-starved for 18 h and either left untreated (lanes 1, 3, 5) or treated with insulin (lanes 2, 4, 6) for 10 min then washed with PBS twice and directly harvested in Leamlli sample buffer. Fifty μg of protein were analysed by SDS–PAGE for phosphorylation of PKB by immunoblotting using an antibody against phosphorylated serine 473. The phosphorylation of this residue activates PKB/Akt. The transfection of wild-type PTEN has little effect, whereas the R234Q mutant induces constitutive phosphorylation of PKB, which cannot be increased further by insulin treatment.

U87MG cells stably expressing either wild-type PTEN or the R234Q mutant were compared with respect to the phosphorylation status of PKB. Phosphorylation of PKB at threonine 308 and serine 473 is indicative for the activity of PKB (reviewed in Chan *et al*, 1999). As reported previously ((Maier *et al*, 1999) and data not shown) parental U87MG cells displayed elevated levels of PKB phosphorylation which could still be further elevated by treatment of cells with insulin for 10 min (see Figure 5

). Stable expression of wild-type PTEN did not significantly alter PKB regulation. In contrast however, expression of the R234Q mutant resulted in a further increase in PKB phosphorylation that could not be induced further by insulin treatment.

## DISCUSSION

Here we report on a novel PTEN mutation that was found as a germline mutation in a patient with meningioma and glioma. To our knowledge this is the first germline mutation reported for brain tumours of multiple lineages. The mutation changes an arginine residue to a glutamine in the C-2 domain of PTEN. This changes the charge of that region (from positive to neutral). Functions of the C-2 region may involve membrane targeting through interactions with membrane phospholipids or association with as yet unknown proteins (Lee, 1999). It has also been shown that the C-2 domain functions to position the phosphatase domain correctly with respect to its substrates. Therefore, mutations in the C-2 domain could alter the overall enzymatic activity of PTEN, without necessarily affecting its phosphatase activity.

We demonstrate that this mutation has functional consequences because the mutant molecules allow cells to proliferate at much higher levels and do not induce or prevent apoptosis. Given that mice with a targeted PTEN mutation on one allele and a wild-type PTEN molecule on the other allele show abnormalities (Di Cristofano *et al*, 1998, 1999), the heterozygous nature of the mutation in the patient does not diminish the importance of the functional findings. The functional consequences of the mutation we have found may help explain why the tumour arose. Of course, it is possible that this mutation has nothing to do with the development of the tumour, or that this is a syndrome with a very low penetrance. However, it seems likely that this mutation also has functional consequences in the tumour. Competition for limited substrates or associated molecules may interrupt normal PTEN function sufficiently to lead to increased proliferation and diminished apoptosis. In fact, the mutant molecule is incapable of inducing apoptosis in BaF/3 cells, which contain endogenous functional PTEN. In some experiments the percentage of apoptotic cells after transfection of R234Q PTEN was even lower than GFP alone, perhaps because endogenous PTEN activity was somewhat inhibited. Another indication that this mutation may not be solely a PTEN null mutant, but can act as a negative competitor, stems from experiments investigating PKB activity. In R234Q PTEN transfectants, PKB activity could not be induced by insulin treatment, in contrast to parental cells or cells transfected with wt PTEN. This result suggests that the R234Q mutation acts to inhibit the residual PTEN activity present in U87MG cells and can therefore be considered as acting as a negative competitor. Thus, the functional consequences of the mutation in transfection studies are consistent with high proliferative activity. Together, these findings suggest that the Arg234Gln missense mutation in PTEN has oncogenic properties.

The main PTEN substrate has proved to be phosphatidyl inositol (3,4,5)-triphosphate (PIP-3) (Myers and Tonks, 1997). Previously, it has been shown that glioma cells lacking wild-type PTEN are characterised by elevated levels of PIP-3 (Li and Sun, 1998). As a consequence, the activity of protein kinase B (PKB/Akt) was found to be elevated, resulting in prevention of apoptosis and increased proliferation. Our results fit this picture in that U87MG cells stably transfected with the R234Q mutant PTEN also show increased proliferation and increased constitutive PKB activity.

One of the main functions of PTEN is to keep PIP-3 levels low. Loss of PTEN function results in increased PIP-3 levels, leading to hyperactive PKB/Akt. The mutation we have found may similarly affect PTEN activity, finally resulting in increased PKB activity, increased cell proliferation, and diminished apoptosis.

**Figure 6:**
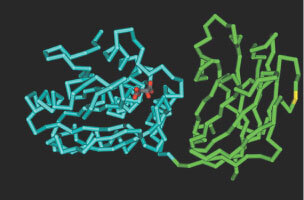
Three-dimensional structure of PTEN, showing the location of the mutation. The mutated amino acid is depicted in yellow in the C-2 domain (green). The substrate binding site of PTEN, located in the phosphatase domain (blue), is shown in red. The model of the PTEN structure is based on Protein Bank code Id5r (Podsypanina *et al*, 1999).

Not many PTEN mutations described in the literature have been associated with the C-2 domain, although Lee *et al* (1999) reported that mutagenesis of the C-2 domain reduced the tumour suppressive activity of PTEN. Our findings are consistent with these results. It is tempting to speculate, based on the X-ray crystallography model of PTEN (see Figure 6

), that the C-2 loop can associate with other proteins. Interestingly the mutation is exactly in the middle of the loop, at a site readily accessible for interaction with other molecules. Future research will undoubtedly identify such C-2 domain-interacting molecules, most likely further explaining the oncogenic nature of the PTEN mutation we have described here. It has been proposed that mutation of basic residues in the C-2 domain reduces the membrane affinity of PTEN and its ability to suppress the growth of glioma tumour cells (Lee *et al*, 1999).

Germline mutations of the PTEN gene are responsible for several inherited conditions, including Cowden disease, Bannayan-Zonana syndrome and Lhermitte-Duclos disease. Here we present a patient with a heterozygous PTEN germline mutation but with no signs of any of these disorders. The patient had an oligodendroglioma, and, in the past, a benign meningioma. The PTEN gene is a tumour suppressor gene and inactivation of both alleles is required for neoplastic transformation. An inherited mutation in one allele with somatic loss of the second allele may result in oncogenesis. Loss of the second allele in human gliomas often occurs by deletion of a part of chromosome 10. However, no LOH could be detected in tumour-derived DNA samples. Inactivation of PTEN by a somatic point mutation could also be excluded, since sequencing of all PTEN coding exons in tumour DNA revealed no changes other than the Arg234Gln germline mutation. One may hypothesise that promoter hypermethylation might function as the second hit at the PTEN gene in the genesis of the patient's tumour. Alternatively, a somatic point mutation could be present in the regulatory sequences of the gene. On the other hand, it has been reported that in a mouse model, inactivation of one PTEN allele has important consequences for cell survival and proliferation (Di Cristofano *et al*, 1999). This suggests that PTEN haplo-insufficiency could be an important factor in cell transformation.
